# Predicting ecological impacts of the invasive brush-clawed shore crab under environmental change

**DOI:** 10.1038/s41598-022-14008-0

**Published:** 2022-06-15

**Authors:** Nora Theurich, Elizabeta Briski, Ross N. Cuthbert

**Affiliations:** 1 GEOMAR Helmholtz-Zentrum Für Ozeanforschung Kiel, Kiel, Germany; 2 School of Biological Sciences, Queen’s University Belfast, Belfast, UK

**Keywords:** Ecology, Invasive species

## Abstract

Globally, the number of invasive non-indigenous species is continually rising, representing a major driver of biodiversity declines and a growing socio-economic burden. *Hemigrapsus takanoi*, the Japanese brush-clawed shore crab, is a highly successful invader in European seas. However, the ecological consequences of this invasion have remained unexamined under environmental changes—such as climatic warming and desalination, which are projected in the Baltic Sea—impeding impact prediction and management. Recently, the comparative functional response (resource use across resource densities) has been pioneered as a reliable approach to quantify and predict the ecological impacts of invasive non-indigenous species under environmental contexts. This study investigated the functional response of *H. takanoi* factorially between different crab sexes and under environmental conditions predicted for the Baltic Sea in the contexts of climate warming (16 and 22 °C) and desalination (15 and 10), towards blue mussel *Mytilus edulis* prey provided at different densities. *Hemigrapsus takanoi* displayed a potentially population-destabilising Type II functional response (i.e. inversely-density dependent) towards mussel prey under all environmental conditions, characterised by high feeding rates at low prey densities that could extirpate prey populations—notwithstanding high in-field abundances of *M. edulis*. Males exhibited higher feeding rates than females under all environmental conditions. Higher temperatures reduced the feeding rate of male *H. takanoi*, but did not affect the feeding rate of females. Salinity did not have a clear effect on feeding rates for either sex. These results provide insights into interactions between biological invasions and climate change, with future warming potentially lessening the impacts of this rapidly spreading marine invader, depending on the underlying population demographics and abundances.

## Introduction

Invasive non-indigenous species (hereafter, “invasive species”) pose a substantial threat to global biodiversity, and the introduction and spread outside their natural range is one defining feature of global change^1,2^. The rate of non-indigenous species introduction has increased sharply in recent decades^3,4^ and exceeds any event in geological history^5^. This increased rate of introduction correlates with globalisation and changes in land use^6–8^. The success of invaders is often dependent on anthropogenic disturbances such as climate change and environmental pollution^1,9–11^. In marine systems in particular, high interconnectedness between habitats through artificial waterways allows non-indigenous species to bypass former biogeographical barriers^12,13^ and exacerbates the risk of invasion and secondary spread. As most studies on invasive species are conducted in terrestrial habitats, there is a paucity of information on invasive species effects in the oceans^14,15^.

Crustaceans in general, and especially predatory brachyuran crabs, are among the most successful groups of marine invaders^16–18^. Prominent invasive species, such as the European green crab *Carcinus maenas* [L.,1758], the Asian shore crab *Hemigrapsus sanguineus* [De Haan, 1853] and the Japanese brush-clawed shore crab *Hemigrapsus takanoi* (Asakura and Watanabe^69^) belong to this group. Despite their important role as a keystone invertebrate group in marine food webs^19^, their introduction into new regions can cause severe ecological and economic impacts on native marine communities^20,21^. Crustaceans appear to be highly resistant to changes related to global warming, such as increased water temperature and ocean acidification, accompanied by a wide salinity tolerance, which could support their invasion success^18^. Decapod crustaceans, such as *H. takanoi*, have several traits which are typical for successful invaders, such as early maturity, high reproductive rates, and reduced cannibalism^22,23^. Thus, they continue to spread, causing ecological and potentially economic impacts^21,24^.

*Hemigrapsus takanoi* became a successful invader in Europe during the last three decades, and most likely reached the Baltic Sea through the Kiel Canal^25^ and ship ballasting operations^26^. The Kiel Canal connects Brunsbüttel at the North Sea and Kiel at the Baltic Sea, and is one of the world’s most frequently used artificial waterways. Each year, more than 30,000 ships use the Canal^27^. *Hemigrapsus takanoi* was first recorded in the Kiel Fjord in 2014. Here, the crab preys on blue mussels (*Mytilus spp*.), a key native species which is also preyed on by native starfish (*Asterias rubens*) and the native crab (*Carcinus maenas*)^25,28^. Variable population demographics have been found for *H. takanoi* in this area, with female *H. takanoi* having a higher abundance, but males being larger overall^28^. Accompanied with larger claws and greater crushing strength, the males consume larger mussels than females, however, it is unclear how sex demographics alter the effect of *H. takanoi* on mussel populations under environmental change.

Today, the Baltic Sea is exposed to many anthropogenically-induced stressors, such as climate change, eutrophication, overfishing, aquaculture, organic pollution, and invasive species^29^. Climate change is one of the driving forces of rapid abiotic regime shifts in the Baltic Sea^30,31^. Due to changes in precipitation patterns, a drop in salinity of about 2 ppt is predicted, and the Baltic Sea will become even fresher by the end of the century^32,33^. Simultaneous warming of about 2–4 °C will exacerbate these abiotic changes^32^. In general, little is known about invasive species impacts as environments shift with climate change^34^. Given the potential risk of *H. takanoi* to the ecosystem, it is pivotal to assess the impact of this invasive species under current and future conditions.

To improve our understanding about combined effects of climate change and invasive species on ecological impacts, this study quantifies the influence of warming and desalination scenarios in the Baltic Sea on the functional response of *H. takanoi.* Functional responses are a common ecological tool to analyse the relationship between resource availability and resource uptake by consumers *per capita*^35,36^. While early studies applied functional responses in community ecology and biological control^36^, they have recently been pioneered as a novel approach in invasion science to quantify the ecological impact of existing and emerging invasive species^37^. The approach also allows for determination of a species’ impact under different environmental contexts, making it suitable for quantification of the effects due to climate change^38^. Specifically, this study aimed to determine what form and magnitude of functional response is exhibited by *H. takanoi* towards *Mytilus* spp. under current and future conditions between sexes. We thus assessed how the consumption rate of *H. takanoi* differed with prey density using functional responses, as well as how falling salinity, increasing water temperature, and sex demographics affect the functional response interactively.

## Results

**Figure 1:**
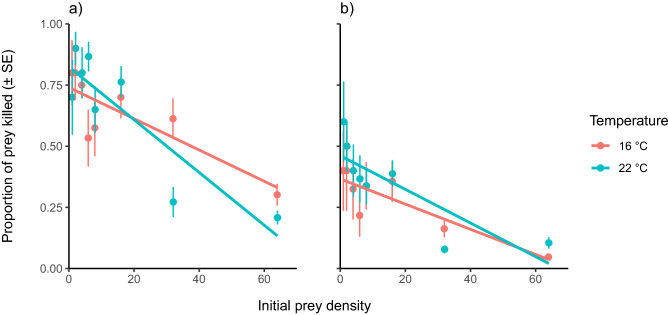
Proportion of killed *Mytilus edulis* across two temperatures, eight prey densities and two sexes by *Hemigrapsus takanoi* (males, **a**; females, **b**). Salinity levels were combined, as salinity was not statistically significant. Points representing the proportion of mean killed prey at each density level ± standard error (SE).

Due to a survival rate of > 99% in the predator-free controls, feeding rates did not need to be corrected for background prey mortality. Thus, the mortality in all treatment groups was attributed to predation, which was frequently observed in situ. Prey consumption rates were significantly different between both sexes overall (Table 2). The 160 female crabs used in the experiment consumed 439 mussels, while the same number of males consumed a total number of 1136 mussels; the males consumed more than 2.5 times than the females overall. The consumption rate was significantly negatively affected by the density of the prey, falling as prey density increased (Table 1; Fig. 1).

**Table 1:** Terms from generalised linear model with a quasibinomial error distribution after Type III analysis of deviance, to determine differences in *Mytilus edulis* prey consumption rates by *Hemigrapsus takanoi* according to “Sex”,” Temperature”, “Density” and “Salinity”.

Predictor	*F* value	df	*p* value
Sex	**51.296**	**1**	**< 0.001**
Temperature	0.010	1	0.919
Density	**198.558**	**1**	**< 0.001**
Salinity	0.031	1	0.861
Sex × Temperature	0.097	1	0.756
Sex × Density	0.000	1	0.985
Sex × Salinity	0.297	1	0.586
Salinity × Density	0.041	1	0.840
Temperature × Density	0.110	1	0.741
Temperature × Salinity	0.111	1	0.740
Sex × Temperature × Density	**4.396**	**1**	**0.037**
Sex × Salinity × Density	0.761	1	0.384
Sex × Temperature × Salinity	0.868	1	0.352
Temperature × Salinity × Density	0.342	1	0.559
Sex × Temperature × Salinity × Density	0.017	1	0.898

Significant terms are in bold.

Moreover, a significant three-way interaction between ‘Sex × Temperature × Density’ was observed (Table 1). This interaction was driven by male feeding rates being significantly temperature-dependent (Fig. 1a), exhibiting a steeper rate of decrease as prey density increased at 22 °C compared to 16 °C (*p* < 0.05). Conversely, female feeding rates were largely temperature-independent as prey density increased (*p* > 0.05) (Fig. 1b). For males, this was driven by a higher feeding rate at low prey densities at the elevated temperature, but inversely a lower feeding rate with warming at the highest prey densities (Fig. 1a). There was no significant difference in consumption rate between salinity groups (Table 1), or between the remaining two-, three and four- way interaction terms (all *p* > 0.05).

**Figure 2:**
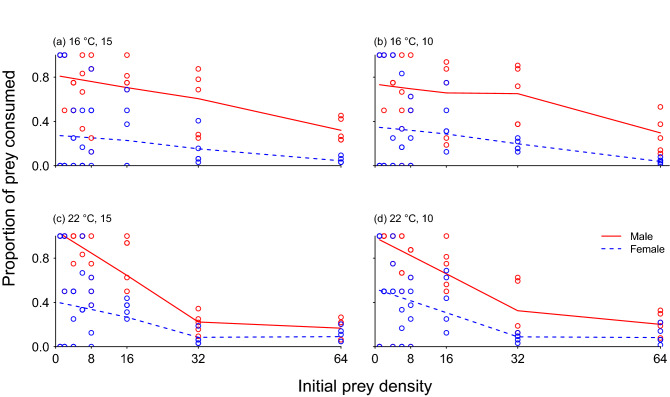
Locally weighted scatterplot smoothing lines fit to the proportion of prey consumed at each *Mytilus edulis* prey density for male and female *Hemigrapsus takanoi* with underlaying data points for (**a**) ambient salinity and ambient temperature, (**b**) desalination and ambient temperature, (**c**) ambient salinity and warming, (**d**) desalination and warming.

All treatment groups displayed significant negative first-order terms, indicating a Type II functional response (Table 2). In addition, the LOWESS trend lines corroborated the decreasing proportion of prey consumed with increasing prey densities for all treatments, thereby confirming Type II functional responses (Fig. 2). In general, the LOWESS trends for females were similar across all abiotic treatment groups, whereas the difference between the two temperature levels was evident for males. Thus, the proportion of prey consumed across prey densities fell more steeply for males at 22 °C (Fig. 2c,d) than at 16 °C (Fig. 2a,b). Salinity did not substantially affect the proportion of prey consumed for either males or females. The curves of males and females were relatively convergent at 22 °C under higher prey densities, but were divergent at low densities (Fig. 2c,d). Contrasting, males and females were more consistently different at the lower temperature across densities (Fig. 2a,b).

**Table 2:** First order terms and significance levels from logistic regressions, with functional response (FR) type, *z* values, first order terms and *p *values for all experimental treatment groups with *Hemigrapsus takanoi* feeding on *Mytilus edulis* prey.

Sex	Temperature (°C)	Salinity	*z* value	First order term	*p* value	FR type
M	16	15	− 8.317	− 0.030	< 0.001	II
M	16	10	− 7.939	− 0.028	< 0.001	II
M	22	15	− 11.400	− 0.047	< 0.001	II
M	22	10	− 9.948	− 0.038	< 0.001	II
F	16	15	− 68.534	− 0.037	< 0.001	II
F	16	10	− 81.984	− 0.044	< 0.001	II
F	22	15	− 63.832	− 0.030	< 0.001	II
F	22	10	− 68.602	− 0.032	< 0.001	II

M and F denote male and female, respectively.

Functional response models consistently returned significant estimates of the attack rate *a* and handling time *h* (Table 3). For both males and females, attack rates trended to increase at higher temperatures. At 22 °C and 15, males exhibited the highest attack rate of all groups, while at 16 °C and 15 females displayed the lowest attack rate overall (Table 3). Conversely, the handling time lengthened at the higher temperature level for males, while the handling time slightly shortened for females at the higher temperature. The shortest handling times were observed within males at 16 °C for both 10 and 15; the longest one was within females at 16 °C and 15 (Table 3). Males thus showed the highest maximum feeding rate at 16 °C, being relatively unaffected by salinity (Table 3). The maximum feeding rate of males at 22 °C was substantially lower (approximately half) than at that at 16 °C. Females, on the other hand, displayed relatively similar maximum feeding rates under all environmental conditions, but these were always substantially lower than the maximum feeding rates of males (Table 3). Similarly, the FRR of males decreased at higher temperatures, whereas it increased for females, thus showing lower and higher impact, respectively. The FRR of males was highest at 16 °C and 15, lowest for males at 22 °C and 10, highest for females at 22 °C and 10 and lowest for females at 16 °C and 15 (Table 3).

**Table 3:** Male (M) and female (F) *Hemigrapsus takanoi* functional response parameter estimates (*a,h*), maximum feeding rate (1/*h*) and functional response ratio (FRR: *a/h*) for all experimental treatment groups towards *Mytilus edulis* prey.

Sex	Temperature (°C)	Salinity	Attack rate (*a*), *p* value	Handling time (*h*), *p* value	Maximum feeding rate (1/*h*)	FRR (*a/h*)
M	16	15	1.584, < 0.001	0.032, < 0.001	31.598	50.036
M	16	10	1.340, < 0.001	0.032, < 0.001	31.365	42.019
M	22	15	3.134, < 0.001	0.080, < 0.001	12.558	39.350
M	22	10	1.971, < 0.001	0.061, < 0.001	16.361	32.242
F	16	15	0.563, < 0.001	0.181, < 0.001	5.518	3.106
F	16	10	0.783, < 0.001	0.168, < 0.001	5.958	4.667
F	22	15	0.813, < 0.001	0.156, < 0.001	6.417	5.215
F	22	10	0.838, < 0.001	0.156, < 0.001	6.409	5.372

**Figure 3:**
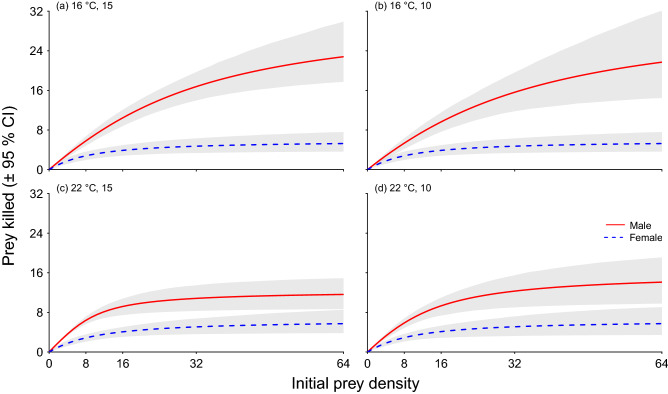
Functional response curves for male and female *Hemigrapsus takanoi* preying upon *Mytilus edulis* under four different abiotic conditions: (**a**) ambient salinity and ambient temperature, (**b**) desalination and ambient temperature, (**c**) ambient salinity and warming, (**d**) desalination and warming. Shaded areas represent bootstrapped (n = 2000) 95% confidence intervals. Lines represent the functional response model (Type II curves for all treatment groups).

Males exhibited significantly higher magnitude functional response curves than females across all treatments, characterised by a clear confidence interval divergence across the prey densities (Fig. 3). Contrastingly, when comparing the confidence intervals of the functional response curves within the sex groups between temperatures, they were mostly convergent (Fig. 3). Only the confidence intervals of males between 16 and 22 °C within the 15 salinity regime were separated at higher densities, indicating a significantly higher feeding rate for males at 16 °C than at 22 °C in ambient salinities.

## Discussion

Predicting ecological impacts of predatory invasive species under environmental change scenarios has remained a major challenge in invasion science^34^. Aquatic ecosystems are at high risk of invasion and their impacts, but many existing and emerging invasive species lack impact assessment^39,40^. Whereas decapod crustaceans are keystone consumers that have precipitated substantial impacts^41^, little is known yet about the predatory impacts of taxa such as the Japanese brush-clawed shore crab *H. takanoi*, which is rapidly spreading across Europe with a relatively recent invasion history. By using comparative functional response approach^37,42^, our study quantified the predatory impacts of male and female *H. takanoi* under warming and desalinisation scenarios, which are expected to increasingly occur in the western Baltic Sea in future^32^. Our study principally determined that the consumption rate of *H. takanoi* strongly depends on the sex, temperature and prey density—with emergent effects among these factors.

Overall, males consumed substantially more mussels than females, irrespective of the environmental treatment. This finding is consistent with previous studies where males’ *per capita* feeding rates exceeded those of females in *H. takanoi*^28,43^, but where different prey densities and environmental contexts were used. This sex-specific difference may emanate from variation in prey preference or handling capacity, with male crabs shown to prefer mussel prey compared to females^44^, whereas females of this species prefer amphipods compared to males^43^. Feeding preferences may also relate to sexual dimorphism between the claws of males and females, as males have larger claws and can exert more force and handle larger prey (with shells) than females, considering their relatively diminutive claws^28^. It is thus possible that the males and females display different prey size preferences based on their crushing strength or claw size, which illustrates the importance of the body size relationship between predator and prey for interaction strength^45,46^. Although male and female crabs were size-matched in our study with regards to carapace width, claws are particularly important in terms of invasion success, with studies on the sibling invasive species *H. sanguineus* showing far greater crushing force than the native *C. maenas*—a mechanism to outcompete similar-sized native species^47^. Hence, it could be possible that the size-matched blue mussels used in the experiment do not correspond with the prey or size preference of females, whereas they correspond to the preference of males based on claw size.

As a poikilothermic organism, the temperature regime was expected to directly impact consumption rates of *H. takanoi*^48–50^. In general, an increase in the feeding rate of animals is estimated at higher temperature levels due to the increase of biochemical and cellular processes until a species-specific thermal optimum^51,52^. However, the results of this study indicate that temperature-dependence is in turn both sex- and density-dependent, with emergent temperature effects between sexes evidenced according to prey density. In this study, the 6 °C warming scenario increased the average maximum feeding rate of females by around 12% overall compared to the average maximum feeding rate at 16 °C. Conversely, the maximum feeding rate of males decreased by about 54% during the 22 °C warming scenario compared to the ambient 16 °C scenario. The feeding rate of males between temperatures was also significantly affected by prey density, unlike that of females. Specifically, at the elevated temperature in males, predation increased towards low prey densities, but feeding rates decreased towards higher prey densities. Feeding rates have been commonly described by a hump-shaped function of temperature, whereby warming increases feeding up to a thermal optimum of a consumer, after which the feeding rate can decrease^53^. The decreased feeding rate of males could therefore have been driven by a thermal stress reaction or by exceeding the temperature optimum, but this interestingly manifested only at higher prey densities. This result contrasts a previous study, albeit where the effects of prey density were not measured, and where a general increase of the feeding rate at higher temperatures was observed^28^.

For both sexes, the consumption rate was fundamentally dependent on the prey density of *M. edulis* and decreased under all abiotic scenarios at higher prey densities. Although trials were performed in the absence of structural complexity, which can influence functional response form^53^, the Type II model was the most appropriate across the four treatment groups. This model predicted high prey consumption rates at low prey densities and a falling feeding rate as density increased. This type of response is also linked with destabilising pressure towards prey populations^37^. While not affecting the functional response type found, temperature significantly affected the functional response parameters, wherein attack rates tended to increase at higher temperatures (i.e. 22 °C). These findings concur with other studies where the attack rate generally increased at higher temperatures until the thermal optima is reached^54^. High attack rates are linked to greater ecological impact^55–57^, but beyond the thermal optima of a species, the functional response may start to decline^58^, due to thermal stress reactions, longer handling times and/or reduced attack rates.

The handling time refers to the time needed by the predator to hunt, process and digest prey^36^ and likewise a shortening of the handling time with increasing temperature is predicted^59,60^. Interestingly, opposing changes in handling time were observed between males and females. Whereas handling time lengthened at the higher temperature level for males, the handling time was slightly shortened for females at the higher temperature level. A similar, unexpectedly longer handling time with warming was observed in a predator–prey system between *Lipophrys pholis* and its amphipod prey *Echinogammarus marinus*^61^. The longer handling time in male *H. takanoi* could again indicate thermal stress, whereas females were not negatively affected by increased temperature. Importantly, the experimental tanks used here were constantly aerated to avoid oxygen limitation, especially at the higher temperature level. This prevented the interacting effects of the decreased capacity of the warmed water to hold dissolved oxygen and concordant physiological insufficiency of the animal’s oxygen uptake^62,63^.

As *M. edulis* only passively escape *H. takanoi* by aggregating with other *M. edulis*, the temperature increase likely did not affect the prey escape tactics, which can be problematic for the predator’s success in other predator–prey systems^60^. Given this is a sessile prey organism, it is also unlikely that temperature influenced encounter rates by altering motility rates of prey, but could have increased foraging behaviours by crabs. The reduced feeding rate of male *H. takanoi* at the higher temperature level was also visible in the reduced FRR, which combines attack rate and handling time and balances the effects of *a* and *h* (i.e. *a/h*)^57^. Contrary to the effect of warming, desalination neither significantly affected the feeding rate nor the functional response parameters in this study. As an euryhaline species, *H. takanoi* can endure a broad salinity spectrum between brackish water and 35 ppt, and therefore a reduction of 5 ppt in salinity did not affect the feeding activity here^64^.

Due to the laboratory-based experimental conditions here, wider community interactions were not captured, such as prey preferences or multiple predator effects^43,65^. It is important to acknowledge that the strict mussel diet used in this experiment therefore does not represent the naturally-broad diet of *H. takanoi*, but as *M. edulis* is a very common resource in the Baltic context, they were an appropriate prey for this single predator–prey functional response experiment. Fundamentally, the use of multiple prey species could mediate prey stability by causing functional responses to shift in form and magnitude (i.e. from Type II to Type III), such as via prey switching at low resource densities^66^. The use of this single prey species nevertheless allowed us to make standardised comparisons according to our research questions and focal variables (temperature, salinity and sex) across prey densities, without additional biotic effects or confounds. Indeed, comparative functional responses typically do not seek to make mechanistic inferences about trophic interactions or the ‘true’ processes influencing their shape (which may require in-field observations), but rather enable standardised comparisons (i.e. phenomenologically) under relevant contexts^37^.

Based on the declining feeding rate, impact is expected to decline under future global warming scenarios for males, but it remains uncertain how the animals behave over a longer period of time acclimated under these conditions, or among different invading populations. Here, the experiment was carried out after one week of acclimatisation and can therefore not provide accurate conclusions about long-term adaptation to changing environmental conditions. Thus, the decrease in feeding rates of males could plausibly be based on a short-term stress reaction that could be mitigated with chronic exposure and adaptation. However, the average maximum feeding rate of males is exceptionally high—almost four times that of the average maximum feeding rate of females. Females were not negatively affected by the temperature regime shift overall, suggesting a different thermal optimum, or a higher ability to endure disturbances, which tends to be a common feature of successful invasive species in general^67,68^. Furthermore, ecological impacts can be assumed to be especially marked through the summer months due to high reported field abundances of *H. takanoi*, which are greatest during their reproductive season in the summer^28^. Notably, whereas these abundance data were obtained from a standardized survey in 2017^28^, based on the numbers of captured *H. takanoi* during the summer of 2021 during our experiment, it can be assumed anecdotally that the population in the sampled area has grown since then (Theurich, personal observation). Accordingly, reductions in *per capita* impact with warming might be compensated by increased predator abundances.

This study provides the first insights into the impact of the invasive crab *H. takanoi* on *M. edulis* using a comparative functional response approach, under conditions expected to be increasingly frequent in the near future. The results of this study clearly demonstrate that *H. takanoi* exhibits a destabilizing Type II functional response under warming and desalinisation scenarios, as predicted for the Baltic Sea. Furthermore, they show that the feeding rate depends on sex, prey density, and temperature interactively, but not salinity. Particularly, higher temperatures reduced the maximum feeding rate of male *H. takanoi* but did not substantially affect the feeding rate of females. Given our results show complex context-dependencies, to make more accurate predictions about the impact of this species in aquatic ecosystems, further research such as functional response experiments with different prey species, different abiotic conditions and long-term population monitoring will be necessary. Overall, this study demonstrates that impact prediction of invasive species under climate change remains a challenge in invasion science, but laboratory experiments can be a useful tool to rapidly assess potential impacts under multiple interacting contexts.

## Material and methods

### Animal collection and maintenance

All animals tested in this experiment were collected in the innermost part of the Kiel Fjord (54°19′44.8″ N 10°08′55.5″ E). The Kiel Fjord is a small arm of the Kiel Bay in the Baltic Sea. The sampling site was characterised by intensively-used harbour buildings with abundant shelter opportunities for crabs. The crabs were collected by dragging-up benthic material with a scrape net (mesh size 0.5 mm), and were separated from other macroinvertebrates, such as the native green crab *C. maenas* and sea star *Asterias rubens* by hand. The water depth at the sampling site was about 2 m, and rich with fine sediment and mussel beds of the key prey item, the blue mussel *M. edulis*. During the sampling period, the water temperature fluctuated between 8 and 15 °C, while the salinity ranged between 12 and 18. The collected individuals were identified based on accepted descriptions, especially by the square shape of the carapace and three lateral spines on the dorsal carapace^69^. Currently there are only two known crab species present in the fjord: *H. takanoi* and *C. maenas*, allowing for unambiguous identification.

Crabs with a carapace size between 14 and 25 mm (mean ± SD: males: 18.10 ± 2.65 mm, females: 16.41 ± 1.90 mm) were collected and sorted by sex, based on their abdominal structure and claw morphology. A further requirement for the crabs used in the experiment was intact chelae and limbs. Therefore, only crabs with both chelae intact and a total number of eight pereiopods were used. Afterwards, the crabs were transported in source water to an environment chamber (16 °C air temperature, 16:8 h light and dark regime) at GEOMAR Helmholtz Centre for Ocean Research Kiel, Germany, and stored in 56 L acclimatisation tanks. The tanks were topped up with filtered Baltic Sea water from the Kiel Fjord. Stones and mussel shells were provided as shelter in each tank as well as continuous filtration. Prior to each feeding experiment, *M. edulis* with a length between 9 and 17 mm (mean ± SD: 12.81 ± 2.21 mm; n = 364, randomly measured out of all sampled mussels; Supplementary Fig. 1) were collected by hand from the same sampling site and stored in the same climate chamber as the crabs in a separate 56 L aerated seawater tank. The size of the mussels was chosen based on a prey selection study by Nour et al.^28^ to match the size preference range for both sexes, and the sizes chosen were balanced among all of the treatments since prey size was not a variable of interest in this study. Nevertheless, this mussel size preference has been shown to differ slightly for males and females, being between 3 and 15 mm for females and 3 and 21 mm for males in the Kiel fjord (Nour et al.^28^). The same prey sizes for each sex were used in our experiment for prey standardisation and predator comparability, and the chosen sizes overlap with the suitable ranges for both sexes.

### Experimental design

Ahead of the experiments, the collected crabs were acclimatised at least for one week to the laboratory conditions (16 °C, 16:8 light and dark regime) under the desired salinity and temperature conditions. The first acclimation tank thus had 16 °C water temperature (i.e. air temperature) and 15 salinity (ambient), the second 16 °C and 10 (desalination), the third 22 °C and 15 (warming) and the fourth 22 °C and 10 (warming and desalination) (Table 4). Temperature in the 22 °C tanks was maintained through heaters (Tetra^®^HT Aquarium Heater) and monitored with temperature loggers (T-logger, HOBO Pendant^®^ Temperature Data Logger). Salinity was monitored using a WTW conductometer (Cond 330i; WTW) and adjusted as needed using dechlorinated tap water. The collected crabs were sorted into each 56 L holding aquaria with a balanced number of males and females. A maximum of 30 crabs was placed in each aquarium to reduce crowding, with crabs fed ad libitum with *M. edulis*. Shelters such as stones and empty shells and air supply were provided in each aquarium. Crab survivability was higher than 98% throughout the experimental and acclimation period. Pilot trials were conducted to evaluate the average consumption rate of the crabs, to inform the appropriate mussel densities used in the feeding experiment for functional responses.

**Table 4:** Abiotic treatment groups used in the functional response experiment.

Treatment group	Ambient	Desalination	Warming	Warming + desalination
Temperature (°C)	16	16	22	22
Salinity	15	10	15	10

Our factorial experiment analysed the functional response of *H. takanoi* under four different abiotic factors for both sexes (Table 4). Therefore, two salinity levels, two temperatures, two sexes and eight different prey densities (1, 2, 4, 6, 8, 16, 32, 64) were crossed in this experiment. Each factor combination was replicated five times. Additionally, three replicates of each factor, except sex, were run predator-free to quantify background prey mortality under each condition (i.e. controls). Our fully factorial experiment thus resulted in a total number of 416 experimental units [(with predators: 5 (replicates) × 2 (temperature) × 2 (salinity) × 2 (sex) × 8 (density)] + [controls: 3 (replicate) × 2 (temperature) × 2 (salinity) × 8 (density)].

In order to measure functional responses under these conditions, plastic aquaria of 2 L capacity (195 × 130 × 117 mm) with an appropriate environment regime and air supply were set up in the same climate chamber. To maintain the correct and constant water temperature in the inner 2 L aquaria, six of these aquaria were placed independently in a water bath of approximately 50 L capacity. Separate water baths were provided for each temperature treatment group to maintain an even temperature across replicates and match holding conditions between ambient and warmed groups. The water baths were filled until the 2 L aquaria volumes were covered ¾. For the warmed groups, a heater (Tetra^®^HT Aquarium Heater) was added as well as a pump for water circulation, to ensure a consistent temperature was applied among the six replicates per water bath. Up to 48 replicates were undertaken each week simultaneously, with treatments (i.e. salinity and sex) randomised within each water bath (i.e. nested within temperature) to eliminate time and positional confounds. The experiment was run over 8 weeks. The background mortality of *M. edulis* was determined using the predator-free controls.

To initiate the trials, crabs were collected from the holding aquaria and placed in the inner plastic aquaria at each treatment, with air supply, and were covered with lids to prevent escape and evaporation. Based on the results of the pilot trials, the crabs were first starved in these aquaria for 48 h to ensure a balanced hunger level of all individuals before feeding. After the starvation period, the water conditions were adjusted if needed. Afterwards, the functional response of *H. takanoi* towards live *M. edulis* was quantified. Before the mussels were added to the tanks with crabs at each density (or without crabs in the case of controls), their size along the anterior–posterior axis was measured with an electronic calliper (TRACEABLE^®^ digital calliper). Afterwards, the crabs were allowed to feed for the following 72 h. After this period, the number of killed mussels was assessed by counting the remaining living prey. *Mytilus edulis* were considered dead when the shells were empty, they did not resist being opened or were floating on the water surface. After the experiment, the carapace size of the crabs was measured with the same electronic calliper to evaluate the average size of the crabs used in this experiment. Each crab was only used once in the experiment to prevent habituation to the experimental conditions.

### Statistics

All statistical analyses were computed using R v. 4.1.0^70^. Significance was defined as a *p* value within the threshold of 0.05 in all analyses. Generalised linear models (GLM) were used to examine consumption rates of *H. takanoi* as a function of temperature, salinity, sex (each two-level factor terms) and prey density (continuous term), and all their interactions. Model residuals were compared to expected simulations via the R package “DHARMa"^71^ to test for overdispersion. Due to residual overdispersion, the quasibinomial family was used for the GLM. Non-significant terms were removed backward, stepwise from the model to obtain the most parsimonious one^72^. Type III analysis of deviance was used to perform F-tests on the final model to compare deviances between the stepwise-reduced models using the “car” package^73^. Further, *post*-*hoc* tests for pairwise comparison were performed using the R package “emmeans”^74^.

The R package “frair”^75^ was used for functional response analyses. Prior to fitting the experimental data to a particular functional response equation, it was necessary to test for the type of functional response. Logistic regressions were thus performed to identify the shape of the functional response curve for each environmental treatment and predator sex, whereby a Type II response is indicated by a significant negative first-order term, and a Type III by a significant positive first-order term then significant negative second-order term^76^. This regression analyses the relationship between the initial prey density and the consumption rate (proportion of prey killed). Furthermore, to visualize the types of functional response, the LOWESS function (locally weighted scatterplot smoothing; smoothing factor 9/10) was fit to the functional response data to illustrate the direction of the proportion of prey consumed in relation to the prey density. Functional responses were modelled for each temperature, salinity and sex treatment using Rogers’ random predator equation, since prey were not replaced as they were consumed^77^, and all treatments were Type II in form (see Results):

1 $$N_{e} = N_{0} \left( {1 - exp\left( {a\left( {N_{e} h - T} \right)} \right) } \right)$$

where *N*_e_ the number of *M. edulis* killed, *N*_0_ is the initial density of prey, *a* is the attack rate, *h* is the handling time and *T* is the total experimental duration (72 h). This equation has been shown to be statistically robust to even total prey depletion across replicates^46^. Non-parametric bootstrapping (*n* = 2000) was used to calculate 95% confidence intervals (CIs) around the functional response curves. This method allows for a visual comparison between the different treatment groups across prey densities, by observing the convergence or divergence in 95% confidence intervals^75^. Attack rate (*a*) and handling time (*h*) estimates were used to calculate maximum feeding rates (1/*h*) and functional response ratios (FRR: *a/h*) over the experimental period^57^, whereby a higher FRR indicates greater ecological impact by synthesising both parameters.

### Ethics approval

Ethical approval for the experimental project was granted by the Ministry for Energy Transition, Agriculture, Environment, Nature and Digitalization of the Federal State of Schleswig Holstein, Germany. All experiments were performed in accordance with relevant named guidelines and regulations. We confirm that the authors complied with the ARRIVE guidelines.

## Supplementary Information

Supplementary Information 1.

Supplementary Information 2.

## Data Availability

Underlying functional response data are available in the supplementary information.
