# Supplementary material for: Predicting ecological impacts of the invasive brush-clawed shore crab under environmental change
Source: Sci Rep. 2022 Jun 15;12:9988. doi: 10.1038/s41598-022-14008-0 (PMC9200808; doi:10.1038/s41598-022-14008-0)
Supplement: Supplementary file 2 — Supplementary Information 2. [file 41598_2022_14008_MOESM2_ESM.pdf]

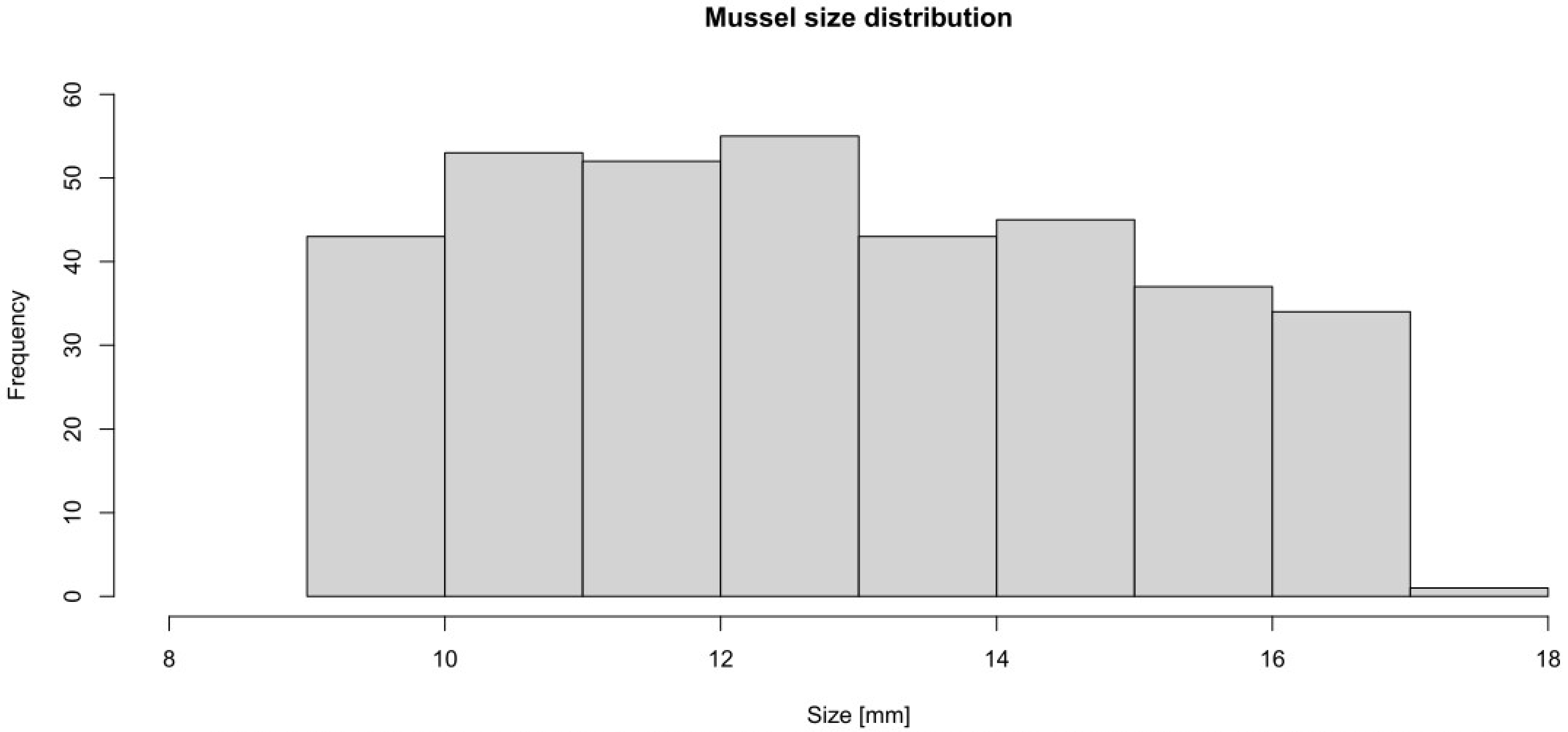

Supplementary Figure 1. Mussel size frequency distribution provided to *H. takanoi* during the functional response experiment
